# Detection of Methylated Septin 9 in Tissue and Plasma of Colorectal Patients with Neoplasia and the Relationship to the Amount of Circulating Cell-Free DNA

**DOI:** 10.1371/journal.pone.0115415

**Published:** 2014-12-19

**Authors:** Kinga Tóth, Reinhold Wasserkort, Ferenc Sipos, Alexandra Kalmár, Barnabás Wichmann, Katalin Leiszter, Gábor Valcz, Márk Juhász, Pál Miheller, Árpád V. Patai, Zsolt Tulassay, Béla Molnár

**Affiliations:** 1 2nd Department of Internal Medicine, Semmelweis University, Budapest, Hungary; 2 Epigenomics AG, Berlin, Germany; 3 Molecular Medicine Research Unit, Hungarian Academy of Sciences, Budapest, Hungary; Sun Yat-sen University Cancer Center, China

## Abstract

**Background:**

Determination of methylated Septin 9 (mSEPT9) in plasma has been shown to be a sensitive and specific biomarker for colorectal cancer (CRC). However, the relationship between methylated DNA in plasma and colon tissue of the same subjects has not been reported.

**Methods:**

Plasma and matching biopsy samples were collected from 24 patients with no evidence of disease (NED), 26 patients with adenoma and 34 patients with CRC. Following bisulfite conversion of DNA a commercial RT-PCR assay was used to determine the total amount of DNA in each sample and the fraction of mSEPT9 DNA. The Septin-9 protein was assessed using immunohistochemistry.

**Results:**

The percent of methylated reference (PMR) values for SEPT9 above a PMR threshold of 1% were detected in 4.2% (1/24) of NED, 100% (26/26) of adenoma and 97.1% (33/34) of CRC tissues. PMR differences between NED vs. adenoma and NED vs. CRC comparisons were significant (p<0.001). In matching plasma samples using a PMR cut-off level of 0.01%, SEPT9 methylation was 8.3% (2/24) of NED, 30.8% (8/26) of adenoma and 88.2% (30/34) of CRC. Significant PMR differences were observed between NED vs. CRC (p<0.01) and adenoma vs. CRC (p<0.01). Significant differences (p<0.01) were found in the amount of cfDNA (circulating cell-free DNA) between NED and CRC, and a modest correlation was observed between mSEPT9 concentration and cfDNA of cancer (R^2^ = 0.48). The level of Septin-9 protein in tissues was inversely correlated to mSEPT9 levels with abundant expression in normals, and diminished expression in adenomas and tumors.

**Conclusions:**

Methylated SEPT9 was detected in all tissue samples. In plasma samples, elevated mSEPT9 values were detected in CRC, but not in adenomas. Tissue levels of mSEPT9 alone are not sufficient to predict mSEPT9 levels in plasma. Additional parameters including the amount of cfDNA in plasma appear to also play a role.

## Introduction

Colorectal cancer (CRC) is the most frequently diagnosed malignant tumor after lung cancer with an incidence of 13.1% in Europe [1]. Screening of CRC is highly cost effective; the cost per life-year saved compares favorably with other preventive treatments, such as therapy of moderate hypertension [2]. The 1-year and 5-year survivals of CRC are 83.2% and 64.3%, respectively [3]. Most long-term survivors of CRC are patients in whom the tumor was diagnosed early, as this offers effective therapeutic inventions for reducing CRC mortality. Early diagnostics should be focused on adenomas since most CRCs evolve on the basis of these premalignant lesions [4].

CRC screening tests currently in use can be divided into two groups: 1) non-invasive tests for primary cancer detection, such as guaiac fecal occult blood test (gFOBT), fecal immunochemical test (FIT) and stool DNA tests; 2) invasive tests that can detect cancer and advanced lesions, such as flexible sigmoidoscopy, colonoscopy, double-contrast barium enema and virtual colonoscopy [5]. However, all of these tests have limitations. Patients' compliance to the non-invasive screening methods is high, but at the cost of relatively lower sensitivity and specificity. CRC-associated mortality can only be reduced by 15–25% using gFOBT, and it detects only 33–75% of CRC [6]. Expensive high quality human hemoglobin-specific FIT detects CRC with a sensitivity of about 60–85% [7]. Furthermore it has a lower prevalence of positives (6.3%) than FOBT (10.3%) [8]. Denters et al. found that 87% of advanced adenomas (larger than 1 cm) can be detected with gFOBT and 75% with FIT. They found that the detection of proximal advanced adenomas is better with FIT compared to gFOBT (27% vs. 17%) [9].

Eighty-five percent of cancerous colonic lesions and 53% of adenomas (size ≥1 cm) can be detected by using stool DNA test (marker panel: methylated vimentin, NDRG4, BMP3, TFPI2 and the mutation marker K-ras). The test has 89% specificity for both lesions [10]. The disadvantage of this test is that it has only a poor acceptance in the general population.

Although invasive colonoscopy has the highest sensitivity and specificity for CRC and adenoma detection, it has the lowest patient compliance rate due to the need of bowel preparation. Additional limitations of this method are the required expertise, as well as higher costs, invasiveness, availability and occasionally adverse events resulting from the procedure. Since the currently established methods for CRC screening either suffer from insufficient effectiveness or from low patient compliance, better and more patient-friendly methods could improve the early diagnosis of CRC.

Blood-based screening techniques offer a new diagnostic tool for benign and malignant colorectal lesions. Wang et al. [11] detected the presence of APC, K-ras and p53 mutations in serum from patients with CRC. They found that these genes may be potential molecular markers for poor clinical outcome of CRC.

Methylated Septin 9 (mSEPT9) was found to be a valuable marker for CRC [12]–[14]. Septin proteins are a group of GTP-binding proteins and belong to a superclass of P-loop GTPases. Septin genes were originally detected in yeast as a critical gene in cell division [15]. They have important role in several cellular processes, such as providing rigidity to the cell membrane, serving as scaffolds to recruit proteins to specific subcellular locales, creating membrane diffusion barriers to establish discrete cellular domains and they play a role in cell polarity determination [15], [16]. The molecular mechanism of Septin 9 (SEPT9) in colon tumorigenesis is still largely unknown; the gene has 18 distinct transcripts generated by alternative splicing and encodes 15 polypeptides and has not been thoroughly studied [17]. This complexity may explain the apparent role of SEPT9 in several diseases, including ovarian and breast cancer [18]–[21], leukemia [22]–[24], urologic cancer [25], [26], brain tumors [27] or CRC [12]–[14], [28]–[33].

Methylated SEPT9 was observed not only in CRC cases, but also in patients with precancerous lesions such as adenomas [30]–[31]. Tänzer et al. detected mSEPT9 in 9% of healthy controls, 29% of precancerous cases and 73% of patients with CRC [30]. In a study by Warren et al. SEPT9 methylation was found in 12% of plasma samples from patients with adenomas [31]. A large prospective study reported recently the suitability of the mSEPT9 test for detecting CRC but insufficient sensitivity (11%) for reliably detecting adenomas [32]. Based on these studies, the mSEPT9 test is suitable for the non-invasive detection of CRC, but does not detect adenomas sensitively.

Johnson DA et al. compared the Septin 9 methylation based blood analysis (Epi *pro*Colon test) with FIT. They concluded that the Epi *pro*Colon test has a similar efficiency for CRC screening as FIT. At a sensitivity of 72.2% Epi *pro*Colon was found to be non-inferior to FIT (68%), albeit it has a lower specificity (80.8% versus 97.4%) [34].

He et al. reported in a recent study the parallel analysis of tissue and peripheral blood samples of CRC patients [33] and they used a multiplex MethyLight assay which included SEPT9. The sensitivities achieved with this assay resulted in similar detection rates of mSEPT9 in tissue (78%) and in plasma (75%). This study, however, did not assess total amounts of cfDNA in plasma or the percent of methylated reference (PMR) values, nor were samples from patients with premalignant adenomas included.

In this study we analysed SEPT9 methylation quantitatively both in plasma and tissue in healthy, adenoma and CRC cases to better understand the correlation between circulating methylated DNA in plasma and their presumed source in tissue. We further used immunohistochemistry (IHC) to compare tissue levels of Septin 9 protein with the presence of mSEPT9 in tissue samples.

## Materials and Methods

### Study design, patients, and lower gastrointestinal endoscopy

A total of 24 healthy controls (no evidence of disease; NED), 26 patients with adenoma with low-grade dysplasia (more than 1 cm diameter or histologically tubulovillous or villous) and 34 patients with CRC (according to the AJCC system: 6 stage I, 11 stage II, 11 stage III, 5 stage IV and 1 unknown) were enrolled in the study (Table 1, S1 Table). The study design was approved by the local ethics committee and government authorities (Regional and Institutional Committee of Science and Research Ethics; TUKEB Nr: 116/2008). Written informed consent was obtained from all patients. Detailed interviews for medical history and physical examinations were performed. All patients included in this study were scheduled for screening colonoscopy for inflammatory bowel diseases or colon neoplasmas. After informed consent, both plasma and tissue samples were taken from the same patients. Exclusion criteria were the following: malabsorption, acute medical conditions, and other malignant diseases (besides colorectal cancer). For detailed clinical and demographic data see Table 2 and S1 Table. During colonoscopy, biopsies were taken for routine histological examination and for study purposes. In the case of adenoma and tumor samples, histological diagnoses were established by pathologists. None of the patients with cancer received chemotherapy, radiotherapy, or surgical invention prior to sampling. Study biopsy samples were stored in RNALater Reagent (Qiagen Inc, Germantown, US) at −80°C until utilization. Peripheral blood samples were taken before colonoscopy using 10 ml EDTA tubes (Vacutainer, Becton Dickinson, New Jersey, USA).

**Table 1:** Overview of disease classifications and number of samples analysed with RT-PCR and IHC.

	NED	Adenoma				Cancer				
**RT-PCR**	**24**	**26**				**34**				
		T	TV	V	NA	I	II	III	IV	NA
		7	15	3	1	6	11	11	5	1
**IHC**	**10**	**14**				**13**				
		T	TV	V	NA	I	II	III	IV	NA
		0	10	2	2	0	7	5	0	1

NED - no evidence of disease (healthy control), T - Adenoma tubulare, TV - Adenoma tubulovillosum, V - Adenoma villosum, NA - not available, I, II, III, IV - Stages according to AJCC system.

**Table 2:** Demographic characteristics of patients.

	NED	Adenoma	Cancer
**Gender (female/male)**	16/8	10/16	19/15
**Age (mean ± SD)**	48±14.9	63.5±11.3	68.3±9.3

NED - no evidence of disease (healthy control), SD - standard deviation, CRC - colorectal cancer.

### DNA extraction, bisulfite treatment and quantitative real-time PCR

Biopsy samples were first subjected to homogenization using a Polytron PT 1600 E benchtop tissue homogenizator (Kinematica Inc., NY, US) to improve yields during DNA extraction. DNA isolation was performed using a High Pure PCR Template Preparation Kit (Roche Diagnostics, Basel, Switzerland) or a QIAamp DNA Mini kit (Qiagen, Hilden, Germany) following the instructions of the manufacturers. DNA was eluted in a final volume of 100 µl and stored at −20°C until processed further. The complete eluates were subjected to bisulfite treatment which was performed in parallel with the plasma samples (see below).

Plasma samples were obtained from 10 ml freshly collected blood samples. Plasma was prepared by two successive centrifugation steps each at 1350 rcf for 12 min. Plasma samples were then either processed directly or were stored at −20° until further use. 3.5 ml of each sample was processed with the Epi *pro*Colon 2.0 Plasma Quick Kit according to the instructions of the manufacturer (Epigenomics AG, Berlin, Germany). Bisulfite converted DNA from both plasma and tissue samples were then analysed using quantitative real-time PCR. A methylated SEPT9 specific fluorescent detection probe, bisulfite-converted unmethylated sequence specific blocker and primers designed in regions lacking CpG dinucleotides were used for PCR reactions (as provided by the Epi *pro*Colon PCR kit). The assay is a duplex PCR determining methylation of SEPT9 and in the same reaction, measuring the total amount of bisulfite converted DNA by using methylation unspecific primer and probes for a beta actin (ACTB) locus. Duplicate PCR reactions were performed on a LightCycler 480 (Roche Diagnostics) instrument.

Since the Epi *pro*Colon test is a qualitative real time assay, we adapted the instructions provided by the manufacturer to record CT values. In addition, in all independent real-time PCR runs, a standard curve was used for quantitative measurements using EpiTect bisulfite converted, fully methylated control DNA (Qiagen Inc, Germantown, US) in concentration steps from 30; 15; 5; 2 to 0.8 ng/PCR (see S2 Table).

### Immunohistochemistry

A section of each tissue sample was subjected to immunohistochemical analysis to detect the presence of Septin 9 protein in these samples. Histologically healthy (n = 10), adenoma (villous and tubulovillous; n = 14) and CRC (stage II and III; n = 13) biopsy samples (Table 1) were fixed in formalin and embedded in paraffin and 4 µm thick tissue sections were cut. After blocking endogenous peroxidase (0.5% hydrogen peroxide and methanol mixture, 30 min, room temperature), antigen retrieval (Target Retrieval Solution 10x concentrate, S1699, Dako, Glostrup, Denmark) was carried out in a microwave at 900 W for 10 min and at 370 W for 40 min. The non-specific binding sites were blocked with 1% human serum albumin (Albumin from human serum, A1653, Sigma-Aldrich, St. Louis, MO, USA, 60 min in room temperature). Immunohistochemical detection of Septin 9 was performed in a humidified chamber using a Septin 9 polyclonal antibody (SEPT9 polyclonal antibody, PAB4799, Abnova, Heidelberg, Germany) in 1∶100 dilution for 60 minutes at 37°C. EnVision + HRP system (Labeled Polymer Anti-Mouse, K4001, Dako) and diaminobenzidine - hydrogen peroxidase - chromogen - substrate system (Cytomation Liquid DAB + Substrate Chromogen System, K3468, Dako) were used for signal conversion. Finally, hematoxylin co-staining was performed (Hematoxylin Solution, GHS132, Sigma-Aldrich). Immunoreactivitiy of Septin 9 protein (Septin-9) was detected with a Panoramic Viewer (Software version: 1.15) digital microscope (3DHISTECH Ltd., Budapest, Hungary) via brightfield whole slide imaging using Panoramic 250 FLASH scanner (3DHISTECH Ltd., Budapest, Hungary) with pco.edge camera (PCO-TECH Inc, Kleinheim, Germany) at 20x magnification.

### Data analysis

Concentrations of mSEPT9 and total amounts of bisulfite converted DNA in each sample were calculated using the established standard curves. Both values were used to calculate the percentage of methylated reference (PMR), expressed as the ratio of mSEPT9 and ACTB, where the amount of ACTB is a proxy measure of the total amount of DNA.

PMR values for each of the three groups were analysed using t-test and ANOVA in combination with Tukey's HSD test to assess statistical significance of the differences. This type of analysis was also applied to assess significance levels for group differences in cfDNA concentrations. Differences were designated as highly significant if p-values were below 0.001 and significant if p-values were below or equal 0.01. The correlation analyses of SEPT9 methylation and cfDNA concentrations were performed using Microcal Origin 6.0 software.

For an additional classification of the samples as either mSEPT9 positive or negative, cut-off levels for PMR values were used. The rationale for this analysis is that the usual application for this biomarker is the detection of either presence or absence of this biomarker in plasma. Cut-off levels were arbitrarily selected based on the calculated PMRs values for both plasma and tissue samples which is further detailed in the Results section below.

The level of Septin-9 protein expression was assessed by applying scores to the intensity of Septin-9 immunohistochemical staining (measured in brightfield on digitalized images). Scores were designated −2 if no immunoreaction was found, 0 if weak, +1 if moderate, and +2 if strong cytoplasmic labelling was observed across the cells analysed. Scores were assigned for 10 healthy, 14 adenoma and 13 tumor specimen. The frequency of the scores were then compared for each of the three groups.

## Results

A total of 84 matching tissue and plasma samples were analysed using a commercially available real time duplex PCR assay which determines in parallel the amount of mSEPT9 and total amounts of DNA in the sample. In this study, this assay has been used to obtain quantitative data to explore potential correlations between the presence of mSEPT9 in tissue and in plasma of the same patients, especially in adenoma patients, since premalignant polyps are of particular interest for the etiology of colon cancer.

### Quantitative DNA determination in tissue and plasma specimens

The total amount of bisulfite converted DNA, as assessed with the ACTB assay, was very different for biopsy specimen and plasma samples. The average size (diameter) of biopsies were 2.3 mm, 3.1 mm, 2.7 mm and the average weight of samples were 3.0 mg, 3.85 mg and 2.8 mg of NED, adenoma and CRC, respectively. The amount of DNA available from biopsies for the analysis were 2.9 µg, 3.3 µg and 2.8 µg for NED, adenoma and CRC, respectively. In contrast to plasma, where identical volumes were subjected to the analysis, the extracted amounts of DNA from the biopsies likely reflect differences in the amount of biopsy material available for experimentation. Plasma samples had much lower amounts of total DNA: mean values were 50 ng/ml, 45 ng/ml and 70 ng/ml for NED, adenoma and CRC, respectively. The DNA detected in plasma predominantly corresponds to the amount of cfDNA in plasma. As identical volumes of plasma were processed from all samples in the three groups, the differences in total DNA amounts detected most likely reflect differences in the amount of cfDNA in these samples. Even though a tendency for higher amounts of cfDNA in CRC was seen, the differences detected between the three groups were statistically not significant.

### SEPT9 methylation in tissue and plasma samples

**Figure 1:**
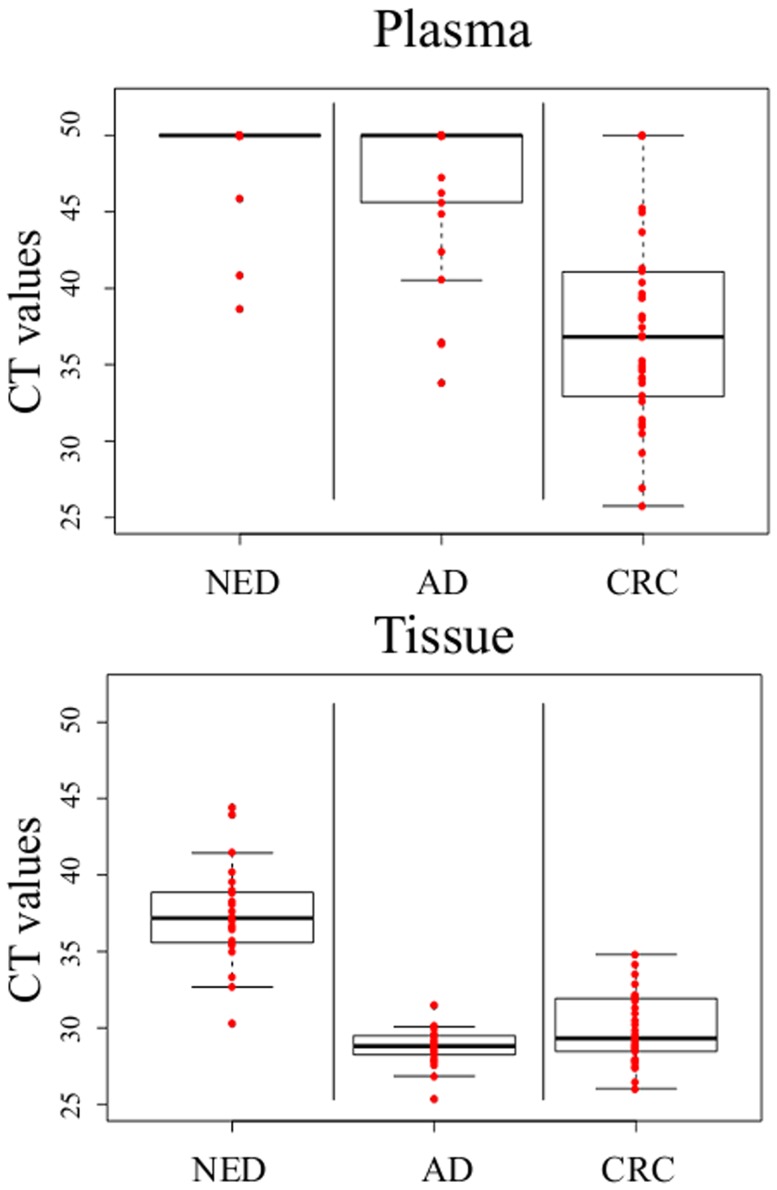
CT values of the assay for mSEPT9 in tissue and plasma samples. Box-plot graphs of CT values for mSEPT9 from healthy (NED - no evidence of disease), adenoma (AD) and cancer (CRC) tissue and plasma samples. The upper and lower edges of each box plot represent the 25th percentile and the 75th percentile, respectively. The line across each box represents the median value for the variable. Individual values are plotted as red dots.

Methylated SEPT9 was detected in all tissue samples (Fig. 1) regardless of the groups, albeit at very different levels. Detection in this case is defined as a CT (cycle threshold) value lower than 50. No significant difference (p = 0.14) was observed for mSEPT9 levels in adenoma and CRC tissue samples, while mSEPT9 levels in the NED tissue samples were much lower, and this difference was highly significant in comparison to either adenoma or CRC (in both comparisons p<0.001).

In plasma, however, mSEPT9 was detected in only a minority of samples from the NED group. Only 3 out of 24 plasma samples in the NED group had CT values below 50, indicative of detectable mSEPT9 levels. In the adenoma group, the number of samples with detectable mSEPT9 levels increased and was highest for CRC patients (Fig. 1, Table 3).

**Table 3:** PMR results for mSEPT9 in plasma and tissue, concentrations of cfDNA detected in plasma samples.

		NED	Adenoma	Cancer
		N = 24	N = 26	N = 34
**Plasma**	Mean PMR (%)^b^	0.01	0.17	5.95
	SD PMR (%)	0.03	0.57	10.92
	Frequency PMR >0.01%	2/24	8/26	30/34
		8.3%	30.8%	88.2%
	Mean cfDNA (ng/ml)	20.52	37.64	70.32
	SD cfDNA (ng/ml)	24.01	27.74	91.47
**Tissue**	Mean PMR (%)^a^	0.52	29.41	21.52
	SD PMR (%)	1.17	20.26	21.74
	Frequency PMR >1%	1/24	26/26	33/34
		4.2%	100%	97.1%

PMR - Percent of methylated reference, SD - standard deviation, NED - no evidence of disease (healthy control), CRC - colorectal cancer.

a - highly significant difference (p<0.001) between NED and adenoma PMR in tissue and between NED and CRC in tissue.

b - significant difference (p = 0.01) between NED and CRC PMR in plasma and significant difference (p<0.01) between adenoma and CRC in plasma.

To be able to directly compare the presence of methylated SEPT9 DNA in tissue and plasma, mSEPT9 levels were expressed as PMR which normalizes the amount of methylated DNA as a ratio to the total amount of DNA measured.

**Figure 2:**
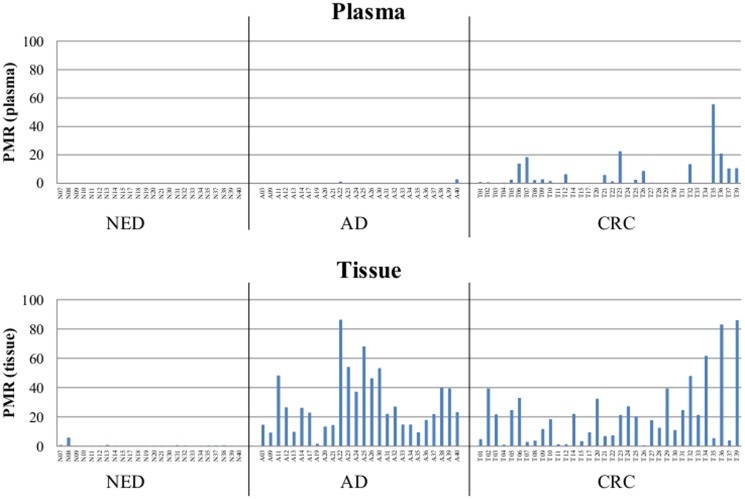
PMR values in plasma and tissue samples. Percent of methylated reference (PMR) of mSEPT9 in healthy (NED - no evidence of disease), adenoma (AD) and cancer (CRC) tissue samples. All tissue and plasma samples are shown individually, and the order of the matching samples within each group is the same. Significance levels for groups comparisons: NED vs. CRC in plasma: p = 0.01; adenoma vs. CRC in plasma: p<0.01; NED vs. adenoma in tissue: p<0.001; NED vs. CRC in tissue: p<0.001.

**Figure 3:**
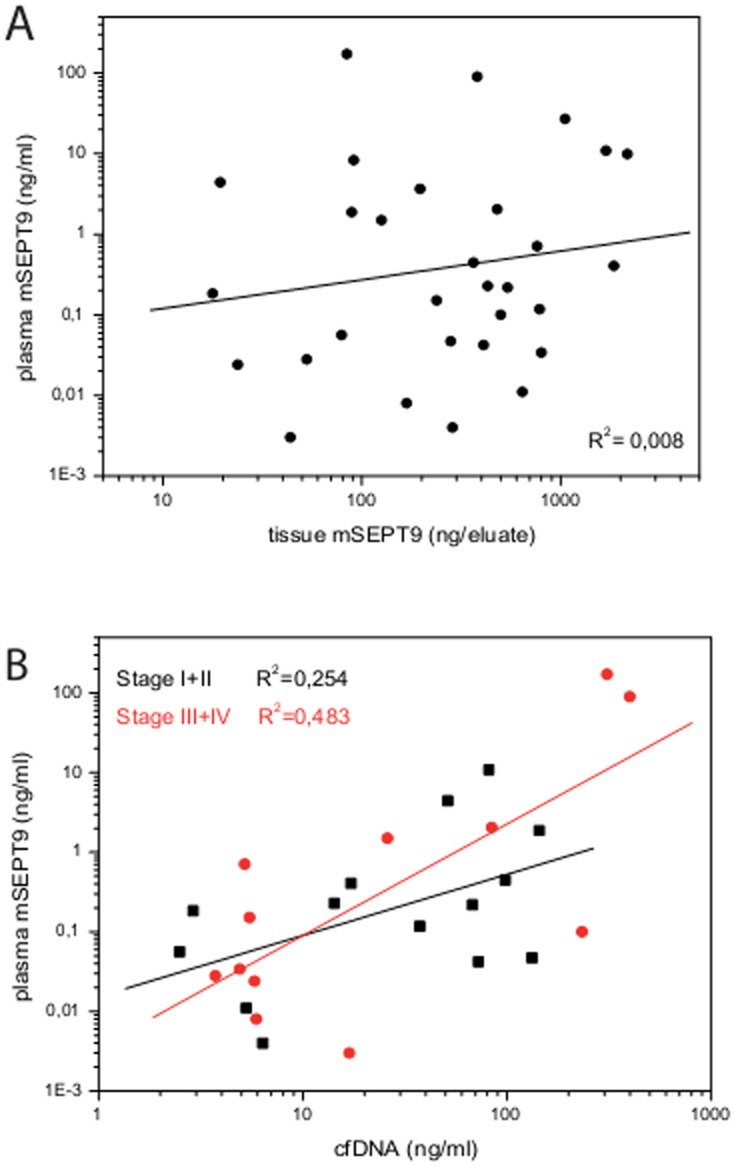
SEPT9 methylation correlation in tissue, plasma and methylation correlation with cfDNA amounts in plasma cases. A, Correlation of mSEPT9 levels between matched SEPT9 positive plasma and tissue cancer samples plotted with logarithmic scales with R^2^ = 0.008. B, Correlation of mSEPT9 levels between matched SEPT9 positive plasma samples from cancer group and cfDNA (circulating cell-free DNA) amounts plotted with logarithmic scales with R^2^ = 0.254 for stage I+II and R^2^ = 0.483 for stage III+IV.

Fig. 2 provides an overview of the calculated PMR values in all three groups and in both tissue and plasma specimens. Only minute levels of mSEPT9 (median of this group: 3.3 ng/biopsy) were measured in biopsies from the NED group, and levels were undetectable in plasma. Significantly elevated levels of mSEPT9 were measured in cancer tissue (median: 372 ng/biopsy) corresponding with well detectable levels of mSEPT9 in the matching plasma samples. Elevated levels were also measured in the adenoma group (median: 531 ng/biopsy), however, a similar correlation was not seen with the matching plasma samples. This indicates that tissue levels of mSEPT9 alone do not determine the level of this biomarker in plasma. Also a case-by-case comparison of mSEPT9 tissue and plasma levels in the CRC group, indicated that the fraction of methylated DNA in tissue is not a good predictor for the amount of this DNA detectable in plasma, which is reflected in a low correlation coefficient (R^2^ = 0.008) as shown in the scatter plot in Fig. 3A.

In an additional qualitative analysis of these data based on counting samples as either mSEPT9 “positive” or “negative”, cut-off levels were chosen for the PMR values. This cut-off was arbitrarily selected at 1% methylation for biopsy samples as the majority of samples in the NED group had PMR values well below this threshold. The chosen cut-off does not represent a threshold based on previous knowledge or a functional correlate but is rather intended to categorize samples with low level methylation from those with clearly elevated methylation levels. Only 1 out of 24 (4.2%) samples in the NED group, for which the mSEPT9 level could be determined reproducibly, was above this level (see Table 3). To be able to also compare biopsy and plasma samples based on this simple classification the same approach was then applied to plasma PMR values. As in plasma samples overall much lower PMR values were detected, corresponding also to much lower levels of DNA present in plasma, a cut-off level at 0.01% PMR was applied for this group. In plasma samples in the NED group mSEPT9 levels above this threshold were detected in only 2 out of 24 (8.3%) subjects, and this corresponds well with the finding in tissue cases. In plasma from adenoma patients this “positivity” was 30.8% (8 out of 26) while all of the tissue samples from these patients were positive for mSEPT9 (26 of 26; 100%) (Table 3). The detection rate of mSEPT9 positive plasma samples in our study was slightly higher compared to sensitivity data for this assay reported previously [13], [14] while the detection of false positives in the healthy group is in the same range as observed in studies with much larger sample numbers [13], [14], [32].

In plasma of CRC patients, the majority of cases (30 out of 34; 88.2%) showed mSEPT9 levels above the 0.01% threshold. While mSEPT9 could be detected in all tissue samples from CRC patients in one case the PMR value was below the 1% cut-off level for tissue; therefore 33 out of 34 (97.1%) of CRC specimens had “positive” mSEPT9 level.

Mean PMR values were calculated for each group (Table 3) and in tissue these were 0.52%, 29.41% and 21.52% for NED, adenoma and CRC, respectively. In plasma mean values were 0.01%, 0.17% and 5.95% for NED, adenoma and CRC, respectively.

Taken together, a high degree of discordance for mSEPT9 levels in tissue and plasma could be observed in the analysed adenoma samples in this study.

### Correlation between mSEPT9 and concentrations of cfDNA

The total amount of circulating cell free DNA was assessed within the same duplex PCR reaction as the amount of mSEPT9. We observed increasing amounts of cfDNA in the three groups although only the difference between CRC and NED was statistically significant (p<0.01, Table 3). The data from two NED and two adenoma cases were excluded from the analysis of cfDNA concentrations as these patients had suspiciously high amounts of cfDNA. Review of patient data indicated that these cases suffered from inflammatory conditions which were undetected at the time of inclusion into the study. For all included subjects, the mean values for cfDNA were for NED 20.52 ng/ml, for adenoma 37.64 ng/ml and for CRC 70.32 ng/ml.

We next analysed whether plasma levels of mSEPT9 were correlated to the total amount of cfDNA, since tumor derived DNA represents a fraction of the total cfDNA. Only tumor cases that were positive for mSEPT9 in plasma were included in this analysis. A stronger correlation (R^2^ = 0.41) was found between plasma mSEPT9 levels and cfDNA levels as compared to the non-correlating data between mSEPT9 levels in tissue and plasma (R^2^ = 0.008, Fig. 3A). This degree of correlation, however, is not very stringent. To further explore which factors may impact the level of plasma mSEPT9 we also analysed the correlations separately for AJCC stages (as provided in S1 Table). The correlations between plasma mSEPT9 and cfDNA for AJCC stages I, II and III either alone or in combination were all rather low (R^2^<0.4), but it was strong for stage IV (R^2^ = 0.93), even though this result is at best suggestive, since only few tumors of stage IV were included in this study. In the absence of a sufficiently large number of samples for stage IV stage we grouped all CRC samples into either early (stage I+II) or advanced (stage III+IV) cancer stages. Comparing these two groups, early versus late stages, lower (R^2^ = 0.254) or stronger (R^2^ = 0.483) correlations were observed according to the disease progression (Fig. 3B). Since the stronger correlation for the late stage cancer is largely an effect of the stage IV cancers, these results will need to be validated in a sample cohort that includes more stage IV cases. Together, however, these data suggest that the concentration of mSEPT9 biomarker in plasma may correlate with cfDNA concentrations predominantly in metastasizing tumors, but shows only weak correlations in early stage- and non-metastasizing tumors.

### Septin-9 protein expression in epithelial cells

**Figure 4:**
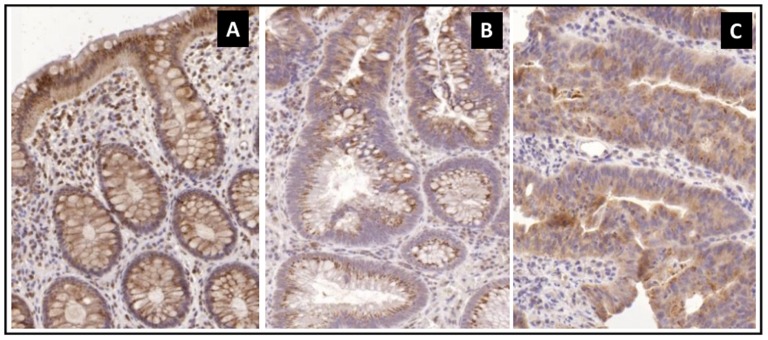
Septin-9 immunohistochemistry in tissue samples. Decreased epithelial expression of Septin-9 protein (brown cytoplasmic immunoreaction) in adenoma (B) and CRC (C) compared to the normal (A) samples (Digital microscope pictures, 20x relative magnification). This observation of Septin-9 protein expression level correlates with previous outcomes [34].

In total, 37 tissue cases of the matched samples were analysed by immunohistochemical staining for Septin-9. A scoring system, in which +2 was assigned to strong cytoplasmic labelling, +1 for moderate and 0 for weak staining, was used to better compare Septin-9 protein expression levels between the analysed groups. In normal samples, diffuse cytoplasmic Septin-9 protein expression was found in epithelial cells, which was more intensive towards the luminal epithelium (typical scoring value: +2; Fig. 4A). The decreased levels of Septin-9 protein expression in tissue samples of adenoma and cancer patients confirmed our findings published in a previous study [35]. In most adenoma samples, moderate or weak immunoreaction localized mainly to the apical cytoplasm of epithelial cells (typical scoring value: +1; Fig. 4B). Septin-9 protein expression was heterogenous in most CRC samples. Weak, diffuse cytoplasmic protein expression was found (typical scoring values: 0 and +1; Fig. 4C), but some parts of the tumor tissue displayed more intensive immunostaining than other areas (see S3 Table).

A scoring system was used to better compare Septin-9 protein expression levels between the analysed groups. Based on this scoring, all specimens of the healthy group (i.e. 100%) received the score +2 indicating a strong immunoreaction for Septin-9 (see S3 Table). The rates of immunoreactive epithelial cells corresponding to score +2 were 42.8% (6 of 14) and 38.4% (5 of 13) in adenoma and cancer, respectively. At the same time epithelial cells which were rated with the scores +1 and 0 were markedly increased in biopsies from adenoma and cancer (S3 Table). Thus a tendency of weakening immunodetection of Septin-9 was observed along the adenoma-carcinoma sequence of disease progression.

## Discussion

In this study SEPT9 methylation was assayed in plasma and matching tissue samples from 84 patients with known or suspected colonic disease. While the detection of mSEPT9 in plasma of patients with colon cancer has been studied extensively [12]–[14], [28]–[32], a quantitative analysis of mSEPT9 levels in matching samples has not yet been reported. We used a commercial duplex assay, which simultaneously detects mSEPT9 and total amounts of DNA in each sample, and analysed these data quantitatively based on calibration curves with known amounts of methylated DNA.

SEPT9 methylation in both adenoma and cancer biopsies was significantly higher compared to the NED group. While individual PMR values for the samples varied considerably, the mean PMR values for the adenoma (29.4%) and CRC groups (21.5%) were comparable. Interestingly, all tissue samples in the NED group were also positive for mSEPT9, albeit at a very low level (mean PMR 0.52%), or approximately 40 fold lower. This observation could suggest that mSEPT9 may also have a physiological role in normal colon tissue possibly by contributing regulatory functions to the proposed involvement of Septin-9 proteins in cytokinesis [15], [16]. Our data also agree with previously published observations of low levels of mSEPT9 positivity in healthy tissue and significantly elevated levels in CRC cases by Lofton-Day et al. [12].

Immunohistochemistry was used to analyse levels of Septin-9 protein in a subset of the tissue samples that were used for mSEPT9 analysis. Comparing the three sample groups, tissue from NED patients showed significantly levels of Septin-9 protein than those from adenoma or cancer, and the detectable protein level in the latter two groups was similarly low.

It is interesting to note that the levels of Septin-9 protein and those of mSEPT9 show an inverse correlation: high levels of Septin-9 protein correspond to low levels of mSEPT9 in the NED group, and vice versa in both adenoma and cancer. This suggests a causal relationship between the methylation status of SEPT9 at this locus and the expression of the protein as had already been suggested in an earlier study [35]. Furthermore, these corresponding data from two different biological levels support the hypothesis that critical molecular changes in colon tissue already emerge during the development of precancerous adenoma, rather than at the onset of CRC.

In contrast to the markedly elevated mSEPT9 levels in adenoma tissue, the matching plasma samples showed only weak levels of mSEPT9 and this indicates a strong difference to the corresponding high mSEPT9 levels in tissue and plasma in CRC samples. Our data on colon polyps are supported by earlier observations which had shown a weak detection of mSEPT9 in plasma from adenoma patients [13], [30], [31]. Warren et al. detected only 12% mSEPT9 positivity in 104 individuals with adenoma, with an overall false-positive rate of 3% using a blood-based test [31]. In another study mSEPT9 showed a sensitivity of 14% for adenomas in plasma samples [36]. Interestingly, a higher detection rate for adenomas based on mSEPT9 analysis was observed depending on the size and type of adenomas [13], [30]. Altogether these different studies suggest that mSEPT9 analysis from peripheral blood is not a sensitive method for the detection of premalignant adenomas. Other non-invasive screening methods like FOBT [9] or stool DNA [10] appear to detect adenomas with a higher sensitivity although the acceptance of these tests compared to blood-based testing methods in the general population is rather low.

The source of cfDNA in peripheral blood has been studied for more than 30 years yet the exact mechanism of its release has still to be elucidated [37], [38]. Several studies reported significant differences in the amount of circulating cell free DNA between disease stages. The cfDNA in patients with CRC was found at levels even about 50 times higher than in healthy subjects [39]. Danese at el. detected significantly higher DNA concentrations in serum not just between controls and CRC patients, but also between controls and adenoma samples [40]. Possible explanations for the wide range of reported cfDNA levels in different studies are physiological factors such as e.g. pregnancy [41], or exhaustive exercise activity [42], [43], or disease specific factors. However, it also reflects methodological differences, such as sample collection and downstream assay differences between the published studies [44]. For instance, there is no general recommendation whether plasma or serum is the better choice for cfDNA detection, although during serum preparation, an increase of cell-free DNA may occur due to lysed lymphocytes [45].

In our study, we detected elevated levels of cfDNA in adenoma and cancer cases as compared to the NED group, while only the difference between CRC and NED reached significance. Within the tumor group there was also a tendency for higher levels of cfDNA with increasing tumor stage, but none of these differences within this group was significant. A recently published study by Danese et al. also investigated the correlation between cfDNA and methylated DNA in plasma of CRC patients and an increase of the absolute concentration of cfDNA with tumor stage was reported [46]. In our study, however, substantial changes in the absolute concentration of cfDNA were predominantly observed for stage IV but less for the other stages, while overall an equally wide range of DNA concentrations was seen in the plasma of cancer patients as in the above mentioned report. With regard to the methylation rate in plasma samples Danese et al. [46] reported elevated rates in the early cancer stages while in our study the highest methylation rates of the SEPT9 biomarker were detected in plasma of late stage cancer patients (i.e. stage IV). This increase in late stage, metastasizing tumors appears plausible as the cancer burden increases, and so does the rate of cell death and the amount of proliferating cancer cells, with a concomitant increase in cfDNA and the portion of DNA derived from tumor cells [47].

Since the absolute amounts of cfDNA are prone to bias for technical reasons (e.g. intact DNA derived from burst lymphocytes during blood sampling might incorrectly increase the levels of plasma cfDNA) such effects would consequently impact the calculated PMR scores (such that, for the above example, the relative amount of tumor derived DNA would be underestimated). Since all blood samples in our study were obtained by the same technical procedure and subjected to the same protocols, and the same is true for the biopsy samples, the comparison of PMR values across the respective groups studied is expected to provide reliable estimates for the amounts of methylated DNA in each group. The comparison of PMR values from plasma and biopsy samples might also be impacted by DNA recovery rates which may differ between plasma and tissue, since cfDNA is mostly of apoptotic origin and therefore is of low molecular weight, while DNA recovered from tissue largely represents high molecular weight. To minimise this potential technical impact in this study DNA from both the plasma and biopsy groups were subjected to the same bisulfite treatments, and no DNA extraction steps were done with the plasma samples.

It certainly will require additional studies to elucidate whether many of the differences detected in independent studies are mainly related to the specific biomarker under investigation or the clinical conditions of the enrolled patients or whether primarily technical aspects account for the heterogeneity of observations.

The discordance between mSEPT9 levels in tissue and plasma in the adenoma group suggests that additional factors than tissue methylation levels are important parameters that determine the amount of DNA from cancer or precursor lesions to be detectable in plasma. Our hypothesis is that poor vascularisation, lower numbers of apoptotic or necrotic cells [47], [48] and additional factors such as high activity of DNase in plasma [49]–[51] are responsible for lower levels of DNA released into the blood stream in adenomas as compared to cancer and that this may be responsible for the different detection levels of SEPT9 in plasma.

## Conclusions

Our analysis of methylated SEPT9 in matching tissue and plasma samples revealed very low levels of mSEPT9 in the tissue of healthy subjects, which may suggest a physiological role of this epigenetic modification also in normal colon tissue. Methylation of SEPT9 measured in plasma samples overall reflected the levels seen in tissue samples in the healthy and tumor group. In contrast, in the adenoma group, elevated mSEPT9 levels in tissue were not associated with increased mSEPT9 levels in the matching plasma samples. This discordance for adenoma is likely due to those factors that impact the release of cellular DNA into circulation. Moreover, also at the level of individual sample pairs tissue levels of mSEPT9 alone are not sufficient to predict the amount of methylated DNA detectable in plasma.

We also observed an inverse correlation between the methylation status of the SEPT9 promoter sequence and the concentration of Septin-9 protein measured by IHC, indicating that expression of this gene may be regulated by DNA methylation.

## Supporting Information

S1 Table **Clinical characteristic of patients.** Macroscopic diagnosis was assigned by gastroenterologist, while microscopic diagnosis was assessed by pathologist. NA - not available, f - female, m - male, npl coli - colon neoplasm.
(DOCX)

S2 Table **Septin-9 scoring in immunohistochemistry.** Scoring of Septin-9 representing the intensity of the immunohistochemical reaction was made on the basis of the following criteria: scoring value was -2 if no immunoreaction was found, 0 if weak, 1 if moderate, and 2 if strong cytoplasmic protein expression was present.
(DOCX)

S3 Table **Calibration curve of standard methylated DNA for A, ACTB (beta-actin) and B, SEPT9 (Septin 9).** Standard curve was used for quantitative measurements using EpiTect bisulfite converted, fully methylated control DNA (Qiagen) in concentration steps from 30; 15; 5; 2 to 0.8 ng/PCR in each RT-PCR run.
(DOCX)
