# Supplementary material for: Detection of Methylated Septin 9 in Tissue and Plasma of Colorectal Patients with Neoplasia and the Relationship to the Amount of Circulating Cell-Free DNA
Source: PLoS One. 2014 Dec 19;9(12):e115415. doi: 10.1371/journal.pone.0115415 (PMC4272286; doi:10.1371/journal.pone.0115415)
Supplement: S1 Table — Clinical characteristic of patients. Macroscopic diagnosis was assigned by gastroenterologist, while microscopic diagnosis was assessed by pathologist. NA - not available, f - female, m - male, npl coli - colon neoplasm. (DOCX) [file pone.0115415.s001.docx]

**Table S1. Clinical characteristic of patients**

Macroscopic diagnosis was assigned by gastroenterologist, while microscopic diagnosis was assessed by pathologist. Mean CTs are represented in the last columns (Plasma, tissue ACTB and SEPT9).

NA - not available, f - female, m - male, npl coli - colon neoplasm

| **Patient code** | **Macroscopic diagnosis** | **Microscopic diagnosis** | **Size** | **AJCC stage** | **Gender** | **Age** | **Plasma** | | **Tissue** | |
| --- | --- | --- | --- | --- | --- | --- | --- | --- | --- | --- |
|  |  |  |  |  |  |  | **ACTB CT** | **SEPT9 CT** | **ACTB CT** | **SEPT9 CT** |
| N7 | negative | negative | NA | NA | f | 55 | 29.99 | 50.00 | 26.88 | 32.66 |
| N8 | negative | negative | NA | NA | m | 68 | 29.77 | 38.62 | 27.26 | 30.28 |
| N9 | negative | negative | NA | NA | m | 29 | 29.96 | 50.00 | 27.99 | 37.18 |
| N10 | negative | negative | NA | NA | f | 70 | 30.22 | 50.00 | 28.51 | 37.58 |
| N11 | negative | negative | NA | NA | f | 46 | 31.26 | 50.00 | 27.89 | 36.58 |
| N12 | negative | negative | NA | NA | m | 57 | 30.59 | 50.00 | 28.76 | 35.73 |
| N13 | negative | negative | NA | NA | m | 60 | 30.26 | 50.00 | 27.67 | 33.29 |
| N14 | negative | negative | NA | NA | f | 30 | 30.55 | 50.00 | 27.31 | 38.95 |
| N15 | negative | negative | NA | NA | f | 74 | 28.74 | 50.00 | 29.00 | 38.79 |
| N17 | negative | negative | NA | NA | f | 60 | 30.46 | 50.00 | 28.72 | 36.58 |
| N18 | negative | negative | NA | NA | m | 56 | 30.11 | 40.84 | 28.66 | 41.44 |
| N19 | negative | negative | NA | NA | f | 56 | 31.02 | 50.00 | 27.76 | 43.93 |
| N20 | negative | negative | NA | NA | f | 39 | 32.86 | 50.00 | 26.90 | 44.42 |
| N21 | negative | negative | NA | NA | f | 43 | 30.44 | 50.00 | 26.57 | 37.18 |
| N30 | negative | negative | NA | NA | f | 42 | 28.47 | 50.00 | 26.66 | 38.25 |
| N31 | negative | negative | NA | NA | f | 42 | 28.71 | 50.00 | 26.56 | 35.00 |
| N32 | negative | negative | NA | NA | m | 32 | 25.57 | 50.00 | 27.11 | 37.27 |
| N33 | negative | negative | NA | NA | f | 17 | 27.58 | 50.00 | 26.18 | 40.17 |
| N34 | negative | negative | NA | NA | m | 42 | 27.97 | 45.83 | 26.45 | 38.07 |
| N35 | negative | negative | NA | NA | f | 67 | 25.12 | 50.00 | 26.43 | 35.38 |
| N37 | negative | negative | NA | NA | f | 37 | 28.09 | 50.00 | 26.81 | 36.43 |
| N38 | negative | negative | NA | NA | f | 57 | 29.11 | 50.00 | 26.58 | 35.42 |
| N39 | negative | negative | NA | NA | m | 45 | 27.42 | 50.00 | 26.60 | 36.97 |
| N40 | negative | negative | NA | NA | f | 29 | 28.59 | 50.00 | 26.77 | 39.56 |
| A3 | polypus coli | adenoma tubulovillosum | >1cm | NA | f | 56 | 31.49 | 50.00 | 27.02 | 28.70 |
| A9 | polypus coli | adenoma tubulovillosum | < 1cm | NA | m | 69 | 27.34 | 36.44 | 27.78 | 30.10 |
| A11 | polypus coli | adenoma tubulovillosum | < 1cm | NA | m | 75 | 28.68 | 44.88 | 27.88 | 27.83 |
| A12 | polypus coli | adenoma villosum | > 1cm | NA | m | 75 | 30.52 | 50.00 | 28.32 | 29.19 |
| A13 | polypus coli | adenoma tubulare | > 1cm | NA | m | 79 | 30.90 | 46.19 | 27.68 | 29.92 |
| A14 | polypus coli | adenoma tubulovillosum | > 1cm | NA | m | 67 | 30.00 | 47.23 | 27.98 | 28.81 |
| A17 | polypus coli | adenoma tubulovillosum | > 1cm | NA | m | 68 | 30.00 | 45.61 | 27.94 | 28.96 |
| A19 | polypus coli | adenoma tubulovillosum | < 1cm | NA | f | 74 | 30.53 | 50.00 | 26.77 | 31.45 |
| A20 | polypus coli | adenoma tubulovillosum | > 1cm | NA | f | 79 | 30.40 | 50.00 | 27.84 | 29.58 |
| A21 | polypus coli | adenoma tubulre | >1cm | NA | f | 60 | 28.02 | 50.00 | 26.49 | 29.51 |
| A22 | polypus coli | adenoma tubulovillosum | >1 cm | NA | f | 63 | 28.17 | 36.37 | 28.23 | 28.55 |
| A23 | polypus coli | adenoma tubulare | >1 cm | NA | m | 63 | 28.40 | 50.00 | 26.88 | 27.58 |
| A24 | polypus coli | NA | >1 cm | NA | f | 55 | 28.02 | 50.00 | 27.02 | 28.47 |
| A25 | polypus coli | adenoma tubulovillosum | >1 cm | NA | m | 60 | 28.39 | 50.00 | 26.67 | 26.86 |
| A26 | polypus coli | adenoma villosum | >1 cm | NA | m | 54 | 27.66 | 50.00 | 25.04 | 25.35 |
| A30 | polypus coli | adenoma villosum | >1 cm | NA | m | 32 | 27.80 | 50.00 | 28.26 | 29.48 |
| A31 | polypus coli | adenoma tubulovillosum | >1 cm | NA | f | 85 | 28.81 | 42.42 | 26.51 | 28.74 |
| A32 | polypus coli | adenoma tubulare | >1 cm | NA | m | 56 | 28.73 | 50.00 | 26.86 | 28.83 |
| A33 | polypus coli | adenoma tubulare | >1 cm | NA | f | 56 | 28.77 | 40.51 | 26.94 | 30.07 |
| A34 | polypus coli | adenoma tubulovillosum | >1 cm | NA | m | 55 | 27.43 | 50.00 | 25.99 | 28.78 |
| A35 | polypus coli | adenoma tubulare | >1 cm | NA | m | 72 | 26.88 | 50.00 | 25.58 | 29.06 |
| A36 | polypus coli | adenoma tubulovillosum | >1 cm | NA | f | 58 | 27.72 | 50.00 | 25.56 | 27.83 |
| A37 | polypus coli | adenoma tubulovillosum | >1 cm | NA | f | 60 | 27.56 | 50.00 | 26.71 | 29.03 |
| A38 | polypus coli | adenoma tubulovillosum | >1 cm | NA | m | 47 | 27.58 | 50.00 | 27.93 | 29.57 |
| A39 | polypus coli | adenoma tubulovillosum | >1 cm | NA | m | 65 | 26.50 | 50.00 | 26.95 | 28.25 |
| A40 | polypus coli | adenoma tubulare | >1 cm | NA | m | 68 | 27.35 | 33.81 | 26.02 | 27.98 |
| T1 | npl coli | adenocarcinoma | NA | III | m | 58 | 30.52 | 37.99 | 28.10 | 31.93 |
| T2 | npl coli | adenocarcinoma | NA | III | m | 77 | 30.02 | 37.46 | 28.20 | 28.48 |
| T3 | npl coli | adenocarcinoma | NA | II | f | 52 | 29.81 | 44.98 | 27.76 | 29.31 |
| T4 | npl coli | adenocarcinoma | NA | III | f | 60 | 30.11 | 43.64 | 28.08 | 33.50 |
| T5 | npl coli | adenocarcinoma | NA | I | f | 84 | 28.14 | 33.75 | 26.54 | 27.41 |
| T6 | npl coli | adenocarcinoma | NA | III | m | 83 | 29.93 | 32.92 | 26.90 | 27.81 |
| T7 | npl coli | adenocarcinoma | NA | NA | f | 68 | 27.94 | 30.46 | 26.50 | 30.49 |
| T8 | npl coli | adenocarcinoma | NA | II | m | 59 | 31.03 | 36.76 | 28.12 | 31.78 |
| T9 | npl coli | adenocarcinoma | NA | IV | m | 60 | 29.85 | 35.22 | 28.19 | 30.21 |
| T10 | npl coli | adenocarcinoma | NA | I | m | 80 | 28.46 | 34.79 | 28.25 | 29.54 |
| T11 | npl coli | adenocarcinoma | NA | II | f | 64 | 30.06 | 50.00 | 26.94 | 32.09 |
| T12 | npl coli | adenocarcinoma | NA | II | f | 82 | 30.81 | 34.95 | 29.18 | 34.17 |
| T14 | npl coli | adenocarcinoma | NA | II | f | 69 | 29.91 | 39.32 | 27.92 | 28.95 |
| T15 | npl coli | adenocarcinoma | NA | III | m | 59 | 28.64 | 45.24 | 29.14 | 32.85 |
| T17 | npl coli | adenocarcinoma | NA | III | m | 73 | 30.21 | 40.35 | 28.64 | 30.90 |
| T20 | npl coli | adenocarcinoma | NA | IV | f | 67 | 28.67 | 50.00 | 28.22 | 28.69 |
| T21 | npl coli | adenocarcinoma | NA | III | f | 85 | 28.96 | 34.60 | 26.80 | 31.33 |
| T22 | npl coli | adenocarcinoma | NA | II | m | 72 | 26.62 | 34.18 | 27.37 | 31.98 |
| T23 | npl coli | adenocarcinoma | NA | IV | f | 66 | 25.23 | 26.95 | 26.85 | 29.26 |
| T24 | npl coli | adenocarcinoma | NA | I | f | 70 | 27.65 | 38.21 | 26.70 | 28.61 |
| T25 | npl coli | adenocarcinoma | NA | IV | m | 70 | 27.35 | 34.02 | 26.45 | 28.83 |
| T26 | npl coli | adenocarcinoma | NA | II | f | 67 | 28.03 | 32.58 | 26.43 | 34.82 |
| T27 | npl coli | adenocarcinoma | NA | I | m | 87 | 27.15 | 36.87 | 26.64 | 29.34 |
| T28 | npl coli | adenocarcinoma | NA | I | m | 62 | 27.56 | 41.26 | 26.02 | 29.13 |
| T29 | npl coli | adenocarcinoma | NA | I | f | 72 | 26.71 | 50.00 | 26.71 | 27.93 |
| T30 | npl coli | adenocarcinoma | NA | II | m | 60 | 26.73 | 41.08 | 26.37 | 29.83 |
| T31 | npl coli | adenocarcinoma | NA | III | m | 56 | 25.96 | 39.66 | 26.67 | 28.75 |
| T32 | npl coli | adenocarcinoma | NA | II | f | 73 | 27.40 | 30.91 | 25.91 | 26.48 |
| T33 | npl coli | adenocarcinoma | NA | II | m | 71 | 28.46 | 39.37 | 25.86 | 27.91 |
| T34 | npl coli | adenocarcinoma | NA | III | f | 71 | 27.37 | 50.00 | 27.17 | 27.73 |
| T35 | npl coli | adenocarcinoma | NA | III | f | 57 | 25.58 | 25.74 | 27.05 | 32.08 |
| T36 | npl coli | adenocarcinoma | NA | III | f | 63 | 26.77 | 29.20 | 27.31 | 27.36 |
| T37 | npl coli | adenocarcinoma | NA | IV | f | 56 | 27.43 | 31.41 | 26.50 | 31.93 |
| T39 | npl coli | adenocarcinoma | NA | II | f | 69 | 27.20 | 31.08 | 26.37 | 26.02 |
