# Supplementary material for: Detection of Methylated Septin 9 in Tissue and Plasma of Colorectal Patients with Neoplasia and the Relationship to the Amount of Circulating Cell-Free DNA
Source: PLoS One. 2014 Dec 19;9(12):e115415. doi: 10.1371/journal.pone.0115415 (PMC4272286; doi:10.1371/journal.pone.0115415)
Supplement: S2 Table — Septin-9 scoring in immunohistochemistry. Scoring of Septin-9 representing the intensity of the immunohistochemical reaction was made on the basis of the following criteria: scoring value was -2 if no immunoreaction was found, 0 if weak, 1 if moderate, and 2 if strong cytoplasmic protein expression was present. (DOCX) [file pone.0115415.s002.docx]

**Table S2. Calibration curve of standard methylated DNA for A, ACTB (beta-actin) and B, SEPT9 (Septin 9)**

**A,**

| **DNA amount per PCR (ng)** | **Log DNA amount** | **CT** |
| --- | --- | --- |
| 30 | 1.4771 | 28.47 |
| 15 | 1.1761 | 29.75 |
| 5 | 0.6990 | 31.05 |
| 2 | 0.3010 | 32.01 |
| 0.8 | -0.0969 | 33.69 |

**B,**

| **DNA amount per PCR (ng)** | **Log DNA amount** | **CT** |
| --- | --- | --- |
| 30 | 1.4771 | 29.04 |
| 15 | 1.1761 | 30.25 |
| 5 | 0.6990 | 31.64 |
| 2 | 0.3010 | 32.99 |
| 0.8 | -0.0969 | 36.33 |
