# Supplementary material for: Detection of Methylated Septin 9 in Tissue and Plasma of Colorectal Patients with Neoplasia and the Relationship to the Amount of Circulating Cell-Free DNA
Source: PLoS One. 2014 Dec 19;9(12):e115415. doi: 10.1371/journal.pone.0115415 (PMC4272286; doi:10.1371/journal.pone.0115415)
Supplement: S3 Table — Calibration curve of standard methylated DNA for A, ACTB (beta-actin) and B, SEPT9 (Septin 9). Standard curve was used for quantitative measurements using EpiTect bisulfite converted, fully methylated control DNA (Qiagen) in concentration steps from 30; 15; 5; 2 to 0.8 ng/PCR in each RT-PCR run. (DOCX) [file pone.0115415.s003.docx]

**Table S3. Septin-9 scoring in immunohistochemistry**

Scoring of Septin-9 representing the intensity of the immunohistochemical reaction was made on the basis of the following criteria: scoring value was -2 if no immunoreaction was found, 0 if weak, 1 if moderate, and 2 if strong cytoplasmic protein expression was present.

| **Score** | **NED** | **Adenoma** | **CRC** |
| --- | --- | --- | --- |
|  | N=10 | N=14 | N=13 |
| +2 | 10 | 6 | 5 |
| +1 | 0 | 8 | 7 |
| 0 | 0 | 0 | 1 |
| -2 | 0 | 0 | 0 |
